# Severe acute kidney injury in COVID-19 patients is associated with in-hospital mortality

**DOI:** 10.1371/journal.pone.0243528

**Published:** 2020-12-09

**Authors:** Jin Hyuk Paek, Yaerim Kim, Woo Yeong Park, Kyubok Jin, Miri Hyun, Ji Yeon Lee, Hyun Ah Kim, Yong Shik Kwon, Jae Seok Park, Seungyeup Han

**Affiliations:** 1 Division of Nephrology, Department of Internal Medicine, Keimyung University School of Medicine, Daegu, Korea; 2 Keimyung University Kidney Institute, Daegu, Korea; 3 Division of Infectious Diseases, Department of Internal Medicine, Keimyung University School of Medicine, Daegu, Korea; 4 Division of Pulmonology, Department of Internal Medicine, Keimyung University School of Medicine, Daegu, Korea; University of Oxford, UNITED KINGDOM

## Abstract

Although the lungs are major targets for COVID-19 invasion, other organs—such as the kidneys—are also affected. However, the renal complications of COVID-19 are not yet well explored. This study aimed to identify the incidence of acute kidney injury (AKI) in patients with COVID-19 and to evaluate its impact on patient outcomes. This retrospective study included 704 patients with COVID-19 who were hospitalized at two hospitals in Daegu, Korea from February 19 to March 31, 2020. AKI was defined according to the serum creatinine criteria in the Kidney Disease: Improving Global Outcomes (KDIGO) guidelines. The final date of follow-up was May 1, 2020. Of the 704 patients, 28 (4.0%) developed AKI. Of the 28 patients with AKI, 15 (53.6%) were found to have AKI stage 1, 3 (10.7%) had AKI stage 2, and 10 (35.7%) had AKI stage 3. Among these patients, 12 (42.9%) recovered from AKI. In the patients with AKI, the rates of admission to intensive care unit (ICU), administration of mechanical ventilator (MV), and in-hospital mortality were significantly higher than in patients without AKI. Multivariable analysis revealed that old age (Hazard ratio [HR] = 4.668, 95% confidence interval [CI] = 1.250–17.430, *p* = 0.022), high neutrophil-to-lymphocyte ratio (HR = 1.167, 95% CI = 1.078–1.264, *p* < 0.001), elevated creatinine kinase (HR = 1.002, 95% CI = 1.001–1.004, *p* = 0.007), and severe AKI (HR = 12.199, 95% CI = 4.235–35.141, *p* < 0.001) were independent risk factors for in-hospital mortality. The Kaplan-Meier curves showed that the cumulative survival rate was lowest in the AKI stage 3 group (*p* < 0.001). In conclusion, the incidence of AKI in patients with COVID-19 was 4.0%. Severe AKI was associated with in-hospital death.

## Introduction

In late December 2019, a cluster of pneumonia of unknown cause was reported in Wuhan, Hubei province, China [1, 2]. High-throughput sequencing subsequently identified the pathogen causing this acute respiratory illness as severe acute respiratory syndrome coronavirus 2 (SARS-CoV-2) [3]. The World Health Organization (WHO) officially named this newly discovered contagious disease as coronavirus disease 19 (COVID-19) [4]. As the outbreak of COVID-19 spread rapidly worldwide, the WHO declared COVID-19 a global pandemic on March 11, 2020. By June 21, 2020, a total of 8,708,008 confirmed cases were reported worldwide, and the mortality rate was 5.3%. In South Korea, one of the earliest countries to experience an outbreak of COVID-19, 12,421 cases were identified with 280 deaths. A joint committee which comprised of nephrologists from the Korean Society of Nephrology and the Korean Society of Dialysis Therapy was established. The committee announced the clinical practice guidelines for preventing transmission of COVID-19 [5]. In Daegu, an explosive increase of COVID-19 cases was reported after infection was initially identified, and 55.5% of COVID-19 patients in Korea were confirmed.

Although COVID-19 is primarily characterized by acute respiratory illness, it can also involve other organs such as the kidney, heart, digestive tract, and blood [6]. The mechanism of kidney involvement has not been exactly elucidated, but kidney injury in SARS-CoV-2 infected patients is of great concern. In previous reports, the incidence of acute kidney injury (AKI) in patients with COVID-19 ranged widely from 0 to 36.6% [7–10]. Most of the studies were reports from China. Studies from other countries are still lacking. We conducted this retrospective cohort study to identify the incidence of AKI in patients with COVID-19 and to evaluate the clinical characteristics of AKI and its impact on patient outcomes.

## Methods

### Study design and participants

The Institutional Review Board of Keimyung University Dongsan Hospital approved this study (IRB number: 2020-04-045). The requirement of informed consent was waived by the Institutional Review Board because of the retrospective and observational nature of the study. This retrospective study included all patients with COVID-19 who were admitted to Keimyung University Dongsan Hospital and Keimyung University Daegu Dongsan Hospital from February 19 to March 31, 2020. All patients showed positive results on the real-time polymerase chain reaction to diagnose COVID-19 by nasopharyngeal swab. Among the 732 patients identified, we excluded those who were under 18 years (n = 21), underwent maintenance dialysis or kidney transplantation (n = 3), had multiple hospital admissions (n = 2), or had insufficient data (n = 2). Finally, 704 patients were analyzed in this study. Patients who showed negative results twice in the polymerase chain reaction test within 48 hours were discharged. The final date of follow-up was May 1, 2020. We accessed the databases to obtain the data used in this study from April 15 to May 15, 2020.

### Data collection and definitions

We reviewed electronic medical records of the study population to retrieve patients’ demographic characteristics, laboratory findings, and radiologic findings. Age, sex, and comorbidities (hypertension and diabetes mellitus) were included in the demographic characteristics. Laboratory findings included complete blood count with differential count, sodium, potassium, blood urea nitrogen, creatinine (Cr), estimated glomerular filtration rate (eGFR), albumin, C-reactive protein, creatine kinase (CK), and lactate dehydrogenase. The eGFR was calculated with the Chronic Kidney Disease Epidemiology Collaboration formula [11]. Old age was defined as patients aged 65 years or older [12]. Pulmonary infiltration on chest X-ray was defined as an increased opacity, excluding pulmonary edema and old pulmonary tuberculosis, as identified by a radiologist.

### Outcomes

AKI was identified according to the serum Cr criteria in the Kidney Disease: Improving Global Outcome (KDIGO) guidelines [13]. AKI was defined as an absolute increase in serum Cr by ≥0.3 mg/dL within 48 hours or increase in serum Cr ≥1.5 times higher from the baseline value within seven days. AKI was staged for severity according to the following KDIGO criteria: stage 1-an increase in the baseline serum Cr to ≥0.3 mg/dL within 7 days or 1.5 to 1.9 times higher within seven days; stage 2-an increase to more than 2.0 to 2.9 times from the baseline serum Cr within seven days; stage 3-an increase to more than 3.0 times from the baseline serum Cr within seven days or increase in serum Cr to ≥4.0 mg/dL initiation of renal replacement therapy. We considered the baseline serum Cr value as the serum Cr level that was measured on the day of admission. We used the highest serum Cr level after AKI detection to identify the stage of AKI on the basis of KDIGO criteria. Severe AKI was considered as stage 2 or stage 3 AKI. We also assessed AKI recovery. AKI recovery was defined as a decrease of the serum Cr value to a value less than 1.20 times the baseline serum Cr level [14]. Other clinical outcomes included renal replacement therapy in-hospital days, intensive care unit (ICU) admission, mechanical ventilator (MV) administration, hospital stay, and in-hospital mortality.

### Statistical analysis

Continuous variables were presented as the mean ± standard deviation. Categorical variables were described as numbers and percentages of patients. The independent t-test or Mann-Whitney test was used to analyze the intergroup comparison of continuous variables. Pearson’s chi-squared test or Fisher’s exact test was performed to compare categorical data. Cox proportional hazard regression analysis was used to identify the independent associations between AKI and in-hospital mortality in patients with COVID-19. The Kaplan-Meier method was used to assess cumulative rates of survival and the log-rank test was used to calculate statistical significance. A *p* value < 0.05 was considered statistically significant. We conducted all statistical analyses using SPSS version 20.0 software (SPSS Inc., Chicago, IL, USA).

## Results

### Baseline characteristics of the study population

A total of 704 patients were included in the present study. The mean age was 57.7 ± 17.6 years, and 210 (29.8%) patients were males. Among these patients, 226 (32.1%) had hypertension and 123 (17.5%) had diabetes mellitus. The mean eGFR was 91.6 ± 24.2 mL/min/1.73 m^2^ and 9.7% of patients had eGFR below 60 mL/min/1.73 m^2^. Among 704 patients, 28 patients (4.0%) developed AKI. Table 1 summarizes the comparison of baseline characteristics between the AKI and non-AKI groups. Compared with the non-AKI group, patients with AKI were older, predominantly males, and had higher rates of hypertension, diabetes mellitus, and chest X-ray infiltration. More patients received antibacterial therapy and lopinavir/ritonavir in the AKI group than in the non-AKI group. The levels of white blood cells, blood urea nitrogen, creatinine, and c-reactive protein were significantly higher in the AKI group than in the non-AKI group. Patients with AKI had lower levels of hemoglobin, platelet, sodium, and eGFR compared with those without AKI.

**Table 1:** Comparison of baseline characteristics.

	AKI (n = 28)	No AKI (n = 676)	*P* value
Age (years)	74.3 ± 12.1	57.0 ± 17.4	< 0.001
Male patient (%)	16 (57.1)	194 (28.7)	0.001
Hypertension (%)	22 (78.6)	204 (30.2)	< 0.001
Diabetes mellitus (%)	16 (57.1)	107 (15.8)	< 0.001
White blood cells (x10^3^/μL)	7.53 ± 4.77	5.4 ± 2.0	0.023
Neutrophil (%)	70.2 ± 12.4	58.3 ± 13.1	< 0.001
Lymphocyte (%)	20.3 ± 10.6	31.1 ± 11.6	< 0.001
Hemoglobin (g/dL)	11.8 ± 1.9	12.6 ± 1.5	0.007
Platelet count (x10^3^/μL)	179.5 ± 85.2	233.2 ± 82.2	0.001
Sodium (mmol/L)	138.0 ± 3.6	140.1 ± 3.6	0.003
Potassium (mmol/L)	4.4 ± 0.8	4.1 ± 0.5	0.108
Blood urea nitrogen (mg/dL)	27.6 ± 21.0	14.5 ± 6.3	0.003
Creatinine (mg/dL)	1.6 ± 1.1	0.8 ± 0.3	0.001
eGFR (ml/min/1.73 m^2^)	56.3 ± 30.8	93.1 ± 22.7	< 0.001
eGFR < 60 ml/min/1.73 m^2^	16 (57.1)	52 (7.7)	< 0.001
Albumin (g/dL)	3.4 ± 0.5	4.2 ± 2.9	0.147
C-reactive protein (mg/dL)	7.9 ± 6.9	2.0 ± 4.0	< 0.001
Creatine kinase (U/L)	93.7 ± 81.1	103.1 ± 291.6	0.872
Lactate dehydrogenase (U/L)	840.6 ± 916.4	487.8 ± 181.0	0.061
Chest X-ray infiltration (%)	24 (85.7)	335 (49.6)	< 0.001
Medication (%)			< 0.001
None	0 (0)	204 (30.2)	0.001
Lopinavir/ritonavir	21 (75.0)	243 (35.9)	< 0.001
Hydroxychloroquine	7 (25.0)	229 (33.9)	0.330

Values are presented as mean ± standard deviation or number (%).

AKI, acute kidney injury; eGFR, estimated glomerular filtration rate.

### AKI and clinical outcomes

During hospitalization, 28 (4.0%) patients developed AKI. Among these patients, 15 (53.6%) were found to have AKI stage 1, 3 (10.7%) had AKI stage 2, and 10 (35.7%) had AKI stage 3 (Table 2). Renal replacement therapy (RRT) was applied to 8 patients and all of them received continuous RRT (CRRT). Twelve (43%) patients achieved recovery of AKI. However, no patient recovered from AKI stage 3. Among the 28 patients, 13 (46%) died. The incidence of in-hospital mortality in patients with AKI stage 3 was 90% and one who survived was still receiving CRRT.

**Table 2:** Characteristics of patients with AKI.

	AKI (n = 28)
AKI	
Stage 1	15 (54)
Stage 2	3 (11)
Stage 3	10 (36)
Severe AKI	13 (46)
Renal replacement therapy	8 (29)
AKI recovery	
Stage 1	10/15 (67)
Stage 2	2/3 (67)
Stage 3	0/10 (0)
In-hospital mortality	
AKI stage 1	3/15 (20)
AKI stage 2	1/3 (33)
AKI stage 3	9/10 (90)

Values are expressed number (%).

AKI, acute kidney injury.

Table 3 shows AKI characteristics according to eGFR at the time of admission. AKI was more frequent in patients in the eGFR < 30 mL/min/1.73 m^2^ group compared with those in the eGFR 30–60 mL/min/1.73 m^2^ group, or the eGFR > 60 mL/min/1.73 m^2^ group (*p* < 0.001). Patients with eGFR < 30 mL/min/1.73 m^2^ had higher incidences of severe AKI and proportion of renal replacement therapy compared to the other groups. In patients with AKI, there were no significant differences of AKI recovery and in-hospital mortality among the groups.

**Table 3:** Characteristics of AKI according to eGFR.

	eGFR ≥ 60 (n = 636)	eGFR 30–60 (n = 54)	eGFR < 30 (n = 14)	*P* value
AKI (%)	12 (2)	9 (17)	7 (50)	< 0.001
Stage 1	8 (1)	3 (6)	4 (29)	
Stage 2	0 (0)	3 (6)	0 (0)	
Stage 3	4 (1)	3 (6)	3 (21)	
Severe AKI (%)	4 (1)	6 (11)	3 (21)	< 0.001
Renal replacement therapy (%)	3 (1)	3 (6)	2 (14)	< 0.001
AKI recovery (%)	6/12 (50)	5/9 (56)	1/7 (14)	0.220
Stage 1	6/8 (75)	3/3 (100)	1/4 (25)	
Stage 2	0/0	2/3 (67)	0/0	
Stage 3	0/4 (0)	0/3 (0)	0/3 (0)	
In-hospital mortality (%)	5/12 (42)	4/9 (44)	4/7 (57)	0.891
AKI stage 1	1/8 (8)	0/3 (0)	2/4 (50)	
AKI stage 2	0/0	1/3 (33)	0/0	
AKI stage 3	4/4 (100)	3/3 (100)	2/3 (67)	

Values are expressed number (%).

AKI, acute kidney injury; eGFR, estimated glomerular filtration rate.

Table 4 shows clinical outcomes according to AKI status. The rates of admission to ICU (*p* < 0.001), administration of MV (*p* < 0.001) and in-hospital mortality (*p* < 0.001) were significantly higher in the AKI group than in the non-AKI group.

**Table 4:** Clinical outcomes of study population according to AKI status.

	AKI (n = 28)	No AKI (n = 676)	*P* value
ICU admission (%)	15 (53.6)	31 (4.6)	< 0.001
Mechanical ventilator administration (%)	13 (46.4)	8 (1.2)	< 0.001
In-hospital mortality (%)	13 (46.4)	11 (1.6)	< 0.001

Values are expressed as means ± SDs or number (%).

AKI, acute kidney injury.

### Impact of AKI on the mortality of patients with COVID-19

Cox regression analyses were performed to identify independent risk factors for in-hospital mortality (Table 5). In the crude analysis, old age (≥ 65 years), male sex, hypertension, diabetes mellitus, high neutrophil-to-lymphocyte ratio (NLR), eGFR < 60 mL/min/1.73 m^2^, low level of albumin, severe AKI, high levels of blood urea nitrogen, c-reactive protein, CK, and lactate dehydrogenase were associated with an increased risk for in-hospital mortality. In the multivariable analysis, old age (HR = 4.668, 95% CI = 1.250–17.430, *p* = 0.022), high NLR (HR = 1.167, 95% CI = 1.078–1.264, *p* < 0.001), elevated CK (HR = 1.002, 95% CI = 1.001–1.004, *p* = 0.007), and severe AKI (HR = 12.199, 95% CI = 4.235–35.141, *p* < 0.001) demonstrated an increased risk for in-hospital mortality.

**Table 5:** Multivariable Cox proportional hazard model to identify risk factors for in-hospital mortality.

Variables	Univariable			Multivariable		
	HR	95% CI	*P* value	HR	95% CI	*P* value
Age ≥ 65 (years)	11.507	3.431–38.597	< 0.001	4.668	1.250–17.430	0.022
Male sex	3.580	1.589–8.066	0.002	1.062	0.356–3.166	0.914
Hypertension	6.026	2.391–15.186	< 0.001	1.588	0.452–5.581	0.470
Diabetes mellitus	7.100	3.103–16.249	< 0.001	2.470	0.900–6.783	0.079
Neutrophil/lymphocyte ratio	1.223	1.162–1.286	< 0.001	1.167	1.078–1.264	< 0.001
Blood urea nitrogen (mg/dL)	1.075	1.055–1.095	< 0.001	0.990	0.936–1.048	0.738
eGFR < 60 (ml/min/1.73 m^2^)	10.255	4.583–22.949	< 0.001	1.318	0.352–4.932	0.682
Albumin (g/dL)	0.099	0.053–0.183	< 0.001	0.378	0.120–1.191	0.097
C-reactive protein (mg/dL)	1.141	1.098–1.185	< 0.001	0.898	0.793–1.016	0.089
Creatine kinase (U/L)	1.002	1.000–1.003	0.006	1.002	1.001–1.004	0.007
Lactate dehydrogenase (U/L)	1.002	1.001–1.002	< 0.001	1.001	1.000–1.003	0.104
Severe acute kidney injury	38.355	16.964–86.721	< 0.001	12.199	4.235–35.141	< 0.001

**Fig 1:**
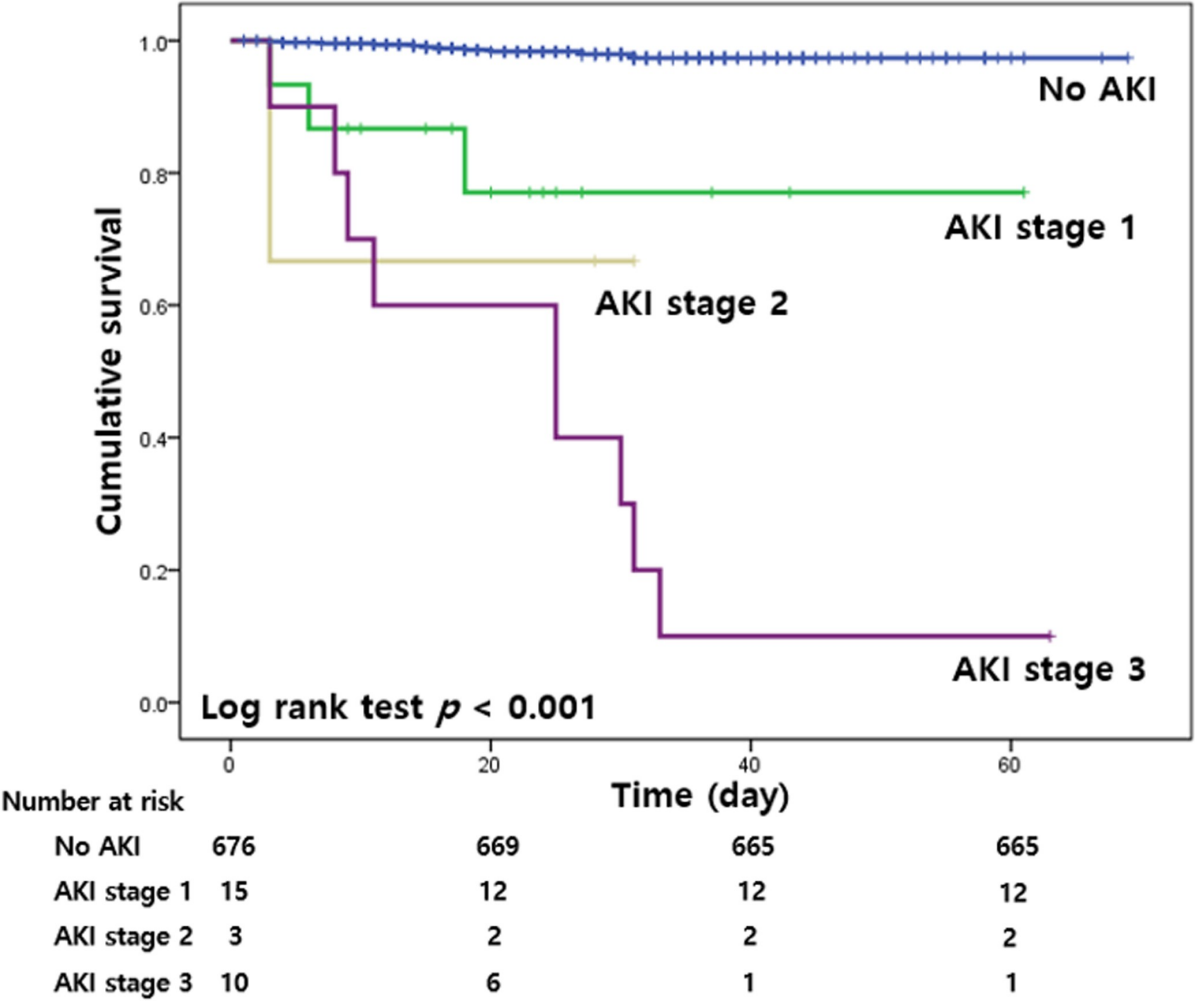
Kaplan-Meier curves for in-hospital mortality according to the stages of AKI. AKI, acute kidney injury.

Fig 1 shows the Kaplan-Meier curves for in-hospital mortality according to the stages of AKI. The cumulative survival rate was lowest in the AKI stage 3 group (*p* < 0.001).

## Discussion

In the present study, we evaluated the incidence of AKI in COVID-19 patients and its impact on outcomes. We analyzed 704 patients with COVID-19 who were admitted to a tertiary care hospital for treatment of severe to critically severe illness, and a community hospital for treatment of moderately severe illness in Daegu, Korea. We observed that the incidence of AKI was 4.0% and that 12 (42.9%) patients recovered from AKI. ICU admission, MV administration, and in-hospital mortality were significantly higher in the AKI group compared with the non-AKI group. Moreover, patients with severe AKI were at a 12.2 times increased risk for in-hospital mortality.

Although acute respiratory illness was the major feature of COVID-19, SARS-CoV-2 also affected other organs, such as the kidneys, heart, digestive tract, and blood [6]. The exact mechanism of other organ involvement has not yet been clarified. Postulated hypotheses include a coronavirus-induced cytopathic effect or sepsis-induced cytokine storm syndrome [15]. Like SARS-CoV infection, the spike protein of SARS-CoV-2 had a strong binding affinity to angiotensin-converting enzyme 2 (ACE2), a host cell receptor. Spike protein is subsequently activated and allows the coronavirus to release fusion peptides for membrane fusion [16, 17]. Besides alveolar cells in the lungs, the expression of ACE2 has been reported in other organs, such as the kidneys and heart [18]. In the kidneys, podocytes and proximal tubule cells were identified as host cells, which suggests that the kidney is a target of SARS-CoV-2 [17].

In the previous reports, there were more males than females in study cohort [6, 8, 10]. In patients with COVID-19, male sex was strongly associated with higher mortality compared with female sex [19]. Sex differences in immune response against COVID-19 contributed to higher mortality in males than females [20]. However, only 210 of the 704 (29.8%) patients were males in our study cohort. This was related to the epidemiological features of COVID-19 in Korea. An explosive increase in numbers of COVD-19 patients in Korea appeared to be mainly caused by exposure among the members of a religious group mainly in Daegu and neighboring areas [21]. By May 31, 2020, a total of 9789 confirmed cases were reported in Korea, and 3905 (39.9%) patients were males. In the present study, predominance by females may have caused by the explosive outbreak related to a religious group in Daegu.

In this study, the incidence of AKI in patients with COVID-19 was 4.0%. In previous reports, the occurrence of AKI in COVID-19 patients varied from 0 to 36.6% [7–10]. Compared with previous coronavirus infections, SARS-CoV and Middle East respiratory syndrome coronavirus (MERS-CoV), SARS-CoV-2 infection seemed to have a lower rate of AKI. A study of 536 patients with SARS found that 36 (6.7%) patients developed AKI, and a previous study including 30 MERS patients revealed that the incidence of AKI was 26.7% [22, 23]. In the early reports of COVID-19 patients, there were lower incidences of AKI ranging from 0 to 5.1% [7–9]. A systematic review involving 24 studies with 4963 confirmed COVID-19 patients showed that the overall incidence of AKI was 4.5% [24]. These results were consistent with our findings. However, a recent cohort study involving 5,449 patients with COVID-19 in the USA reported that 1,993 (36.6%) patients developed AKI [10]. The discrepancy could not be adequately explained. The South Korean government used therapeutic living centers as isolation facilities because of a rapid increase of COVID-19 patients and a shortage of hospital beds. Asymptomatic to mild COVID-19 patients were quarantined in therapeutic living centers. Moderate cases of COVID-19 were suitable for the community hospital, and severe to critical patients were admitted to a tertiary care hospital [25]. Keimyung University Dongsan Hospital was designated as a tertiary care hospital for the treatment of patients with severe to critical symptoms and Keimyung University Daegu Dongsan Hospital was designated as a community hospital for patients with moderate severity. Therefore, patients with moderate to critical symptoms were included in this analysis. In the present study, the study population was younger and had lower incidences of diabetes mellitus and hypertension than the recent study in the USA. Moreover, less patients (3.0%) received MV in our study compared to those of the recent trial (21.8%). However, these differences could not fully explain the discrepancy, and further clinical trials are warranted.

In patients with AKI, the rates of admission to ICU, administration of MV, and in-hospital mortality were significantly higher compared with those without AKI. These results were in accordance with the findings of previous studies [8–10]. Additionally, we found that higher stages of AKI were associated with a poor clinical prognosis of COVID-19 patients. Of 10 patients who developed stage 3 AKI, 90% died and one was still receiving CRRT. We demonstrated that patients with lower eGFR were more likely to develop AKI during hospital stays, which is consistent with previous report [8]. In this study, patients with lower eGFRs had higher incidences of severe AKI and proportion of renal replacement therapy. Therefore, it is important to monitor kidney function in COVID-19 patients, especially in patients with low eGFRs at the time of admission.

In the present study, 8 of the 28 (28.6%) AKI patients received RRT and all of them required CRRT. The timing of RRT has long been a controversial issue in AKI patients. Theoretically, early initiation of CRRT has beneficial effect on cytokine removal in patients with COVID-19. As many inflammatory factors are medium and high molecular weight molecules, high cut off membrane can increase cytokine removal [26]. Also, polystyrene-based hemoadsorber, such as Cytosorb, can adsorb cytokine and middle molecular weight solutes [27]. However, there are no available data about the timing of CRRT, high cut off membrane and Cytosorb in the setting of COVID-19. In our cohort, all patients who needed CRRT treated with continuous veno-venous hemodiafiltration. Among 8 patients, 1 patient received early CRRT. Seven patients died and 1 patient who initiated CRRT with definite indications was still receiving CRRT. As hypercoagulable state is often induced by COVID-19, anticoagulation strategy is important to reduce CRRT circuit clotting [28]. Regional citrate was more effective anticoagulation modality than other anticoagulants in terms of reducing the risk of circuit clotting [29]. Another study reported that nafamostat mesylate inhibited SARS-CoV-2 infection of lung cells as well as anticoagulant effects [30]. In the present study, 4 of the 8 CRRT patients received concurrent extracorporeal membrane oxygenation. Unfractionated heparin was used in those patients, and nafamostat mesylate was prescribed to patients who only received CRRT. Further clinical trials are needed to evaluate the effect of anticoagulants in COVID-19 patients receiving CRRT.

We also evaluated renal recovery in patients who developed AKI. Of 28 patients with AKI, 12 (42.9%) recovered from AKI. Previous study involving 333 COVID-19 patients revealed that 4 of the 22 AKI patients (18.2%) recovered from AKI [9]. Stage 1 AKI was most frequent, as 53.6% of our population compared with 18.2% of their patients had stage 1 AKI. In our study, 67% (10/15) of stage 1 AKI patients achieved AKI recovery. Additionally, the follow-up duration was longer than in previous studies. These differences may affect the results.

Multivariable analysis identified old age, high NLR, elevated CK level, and severe AKI as independent risk factors for in-hospital mortality. Host immune status was an important factor in viral infection. SARS-CoV-2 was more likely to infect patients with weak immune functions, such as patients of old age. Old age was significantly associated with in-hospital mortality [8, 10]. NLR, easily calculated from complete blood count with differential count, was a widely used marker for a patient’s inflammatory status. Increase of NLR was a risk factor for mortality in not only critical care, but also multiple chronic conditions [31]. In patients with COVID-19, increased NLR was a predictor of severe illness and an independent risk factor for mortality [32]. CK was an important enzyme in tissues which consumed ATP rapidly [33]. Measurement of CK levels was used for damage monitoring in CK-rich tissues, including skeletal muscle, cardiac muscles, and the brain. Increased levels of CK in patients with COVID-19 might be an indicator of respiratory muscle injury. Recent prospective cohort study involving 233 patients showed that an elevated CK level was an independent predictor of mortality in patients with COVID-19 [34]. These results were consistent with our findings. AKI was a common complication in hospitalized patients and a well-known risk factor for in-hospital mortality [35]. Previous reports showed that kidney injury was associated with a poor prognosis among patients with COVID-19 [8–10]. A recent study involving 469 COVID-19 patients in USA revealed that AKI was an independent risk factor for in-hospital mortality [36]. A meta-analysis including 40 studies and 24,527 COVID-19 patients also demonstrated that AKI is associated with higher risk of mortality [37]. In accordance with previous studies, our study demonstrated that patients with severe AKI were at a 12.2 times increased risk for in-hospital mortality, and the cumulative survival rate was lowest in the AKI stage 3.

In this study, there were 3 kidney transplantation recipients excluded from the analysis. Prescribing proper immunosuppressive regimens in transplantation recipients with infection has been a critical issue. Among maintenance immunosuppressive drugs, calcineurin inhibitors (CNIs) were the cornerstone nowadays. Experimental evidence suggested that CNIs, both cyclosporine and tacrolimus, inhibited viral replication of SARS-CoV through the inhibition of peptidyl-prolyl cis-trans isomerases [38, 39]. A study of 14 patients with organ transplantation in Italy showed that clinical course of COVID-19 patients who received CNIs was generally mild and CNIs did not negatively affect the patients' outcome [40]. In our cohort, all patients received CNIs as maintenance immunosuppressants. Two patients were treated with tacrolimus and 1 with cyclosporine. We decided to maintain CNIs and discontinue mycophenolate mofetil. Two patients with tacrolimus developed AKI stage 1, and patients with cyclosporine had no AKI. Two AKI patients achieved AKI recovery and all patients were discharged with favorable outcomes. These results were consistent with previous reports.

The present study has some limitations. First, because of the rapid outbreak of COVID-19 and shortage of medical resources, we could not obtain full laboratory support, in particular the urine test. We could not evaluate the effect of SARS-CoV-2 infection on urinalysis. The lack of data on urine output may lead to the misclassification of AKI. Second, most patients did not have a creatinine level before admission; therefore, we used serum Cr level which was measured on admission as the baseline Cr value. Patients with AKI at the time of admission may not have been included in the AKI group, which may lead to underestimation of the AKI rate. In addition, previous chronic kidney disease was known as a risk factor for mortality in patients with COVID-19 [41]. We could not evaluate the effect of pre-existing kidney disease. Third, we could not collect all confounders influencing the outcome. Therefore, the results may be subject to residual and unmeasured confounders. Fourth, although we enrolled a relatively large number of patients with moderate to critical symptoms in the present study, this was a retrospective cohort study performed in two centers in Korea. Thus, we could not generalize our findings to all COVID-19 patients.

In conclusion, the present study demonstrated that the incidence of AKI in patients with COVID-19 was 4.0%. Kidney involvement was associated with poor prognosis, including admission to ICU, administration of MV, and in-hospital mortality. Additionally, severe AKI was an independent risk factor for in-hospital mortality. In the management of patients with COVID-19, regular monitoring of kidney function should be emphasized, and clinicians must pay attention to AKI. Early detection of AKI and prompt intervention may improve patient outcomes in COVID-19.

## Supporting information

S1 Dataset (XLSX)
